# Application of an Eco-Friendly Adhesive and Electrochemical Nanostructuring for Joining of Aluminum A1050 Plates

**DOI:** 10.3390/ma16062428

**Published:** 2023-03-18

**Authors:** George C. Papanicolaou, Lykourgos C. Kontaxis, Nikolaos Kouris, Diana V. Portan

**Affiliations:** Composite Materials Group, Department of Mechanical Engineering and Aeronautics, University of Patras, Rio, GR 265 04 Patras, Greece

**Keywords:** eco-friendly adhesive, aluminum A1050, single-lap joint (SLJ), tensile-shear testing, three-point bending, electrochemical anodization, alumina nanotubes

## Abstract

In adhesive joints used in several industrial applications, the adherends’ bonding is made using an adhesive, which is usually an epoxy resin. However, since these adhesives are derived from petroleum fractions, they are harmful to the environment, due to the pollutants produced both during their manufacture and subsequent use. Thus, in recent years, effective steps have been made to replace these adhesives with ecological (green) ones. The present work focuses on the study of aluminum A1050 joints bonded with a green adhesive; the study also involves the electrochemical anodization method applied to adherends for nano-functionalization. The nanostructured aluminum adherends allow the formation of an expanded surface area for adhesion, compared to the non-anodized adherends. For comparison reasons, two different adhesives (Araldite LY1564 and Green Super Sap) were used. In addition, for the same reasons, both anodized and non-anodized aluminum adherends were joined with both types of adhesives. The lap joints were subsequently tested under both shear-tension and three-point bending conditions. The major findings were that aluminum A1050 anodization in all cases resulted in shear strength enhancement of the joints, while joints with both aluminum anodized and non-anodized adherends and bonded with the eco-friendly adhesive showed a superior shear behavior as compared to the respective joints bonded with Araldite adhesive.

## 1. Introduction

In industrial load-bearing components manufacture, adhesives are used in joining both similar and dissimilar materials (e.g., metal-to-metal, metal-to-composite, metal-to-rubber, metal-to-glass, metal-to-wood, etc.) together [1]. The use of adhesives for joining materials is targeting to the development of cheaper and lighter products. Adhesive joining is a tried-and-true fastening technique in the industrial industry that expands the present range of traditional joints. Elastic adhesives were first utilized to assemble and join elements of automobiles in the early 1980s. Regardless of whether the joint is elastic or stiff, the adhesive joining method differs fundamentally from mechanical joining techniques. Structural adhesives are frequently used in the industry as joining techniques because flexible adhesives, as opposed to rigid ones, may bend when subjected to stress. Welding issues frequently include deformation and thermal distortion. To fix these flaws, sanding and polishing procedures are used. Moreover, some metals, such as some varieties of ultra-high strength steel, deteriorate in the presence of heat [2]. By more uniformly spreading stresses, adhesive bonding prolongs the useful life of a component or thins out the material. In contrast to stiff structural adhesive-bonded structures, which distribute stress along the bond line, elastic adhesives offer a stress distribution over the whole bonded region.

In addition to joining two parts, adhesives also act as a sealant. The protection against galvanic and crevice corrosion provided by adhesive bonding has been demonstrated to be superior to that of traditional fastening techniques. Unlike mechanical joints, adhesive joints need some time to reach their maximum or ultimate strength. Assembly handling and advancement to the following manufacturing stage, however, typically occurs before assemblies attain their peak performance. Although it is well-established in the industrial sector, structural adhesive bonding has a lot of potential applications. An outdated joining technique is replaced and much improved by adhesive bonding.

However, although adhesive bonding looks like a promising solution for cheap and lighter product manufacture, there are some serious problems that must be overcome. The main problem is that adhesives must fulfill the performance requirements of the product while, at the same time, they must be eco-friendly so that they do not harm the global environment. For example, adhesives based on phthalates and formaldehyde release emissions as they dry, which can significantly affect air quality, resulting in human skin and eye irritation as well as liver and kidney damage. The same, or worse, problems can occur when using other adhesives containing several harmful chemicals [3].

This aspect is becoming more significant while developing novel polymers because of rising environmental consciousness [4,5,6,7]. A rising variety of environmentally friendly epoxies have been created recently, and these materials are now made from natural and renewable resources including epoxidated oils (soybean oil, cottonseed oil, etc.), furans, sorbitol, and others [8,9,10]. Several of these are already offered commercially [11,12]. Depending primarily on their green carbon content, level of toxicity, environmental effect throughout their life cycle (greenhouse emissions, energy consumption, etc.), and end-of-life possibilities, these materials can be eco-friendly (recyclability, biodegradability, compostability). A variety of green materials have been designed and developed to employ as adhesives [13,14,15,16,17,18]. However, further study is required to investigate the environmental impact and enhance the mechanical performance of eco-friendly adhesives.

Depending on which component (i.e., adhesive or adherend) of the lap joint plays the primary role when the joint is loading, one can use adhesives belonging to one of the following four main categories, namely, (a) sealants, (b) low-strength adhesives, (c) medium-strength adhesives, and (d) high-strength adhesives. 

**Figure 1:**
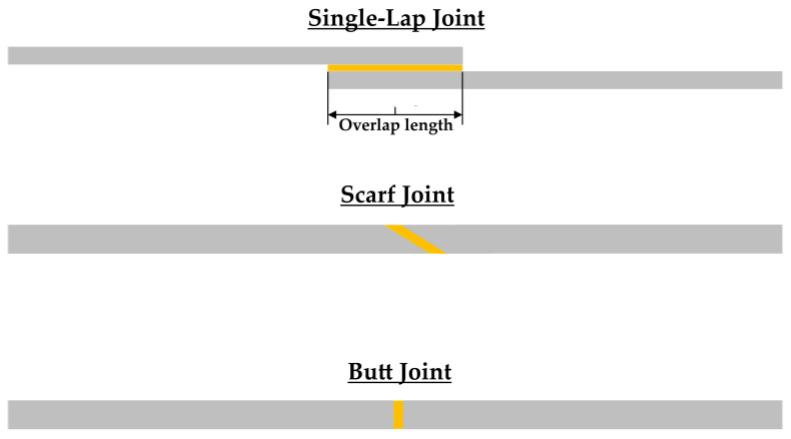
Basic types of adhesively bonded joints.

Adhesive bonding is an interdisciplinary area of research since, depending on the specific application, it combines knowledge coming out from different sciences, such as chemistry, mechanical engineering, medical and medicine, biology, and other sciences. The main areas of application adhesive joints being used comprise aeronautics, aerospace, and medicine. On the base of their geometrical characteristics, adhesively bonded joints, are classified into many categories the basic types of which are (a) single-lap joints, (b) scarf joints, and (c) butt joints (Figure 1). In any case, the choice of the appropriate joining technique is of cardinal importance to lead in a high-strength structure. Each one of the three types of joints mentioned has its own advantages and disadvantages. Single-lap joints (SLJs) are the simplest ones to adhesively join two materials together. In the case of scarf joints, there is an inclined section the slope of which dictates the stress field developed upon loading. Finally, a disadvantage of butt joints is the small overlap area.

There are numerous factors affecting the mechanical performance of adhesive joints. Amongst them, except for the joint geometry and the adhesive type, one can mention the mechanical properties of the adherends/adhesive, the overlap length, the thicknesses of the adherends/adhesive, the adherends’ surface roughness in the overlap area, etc. Testing and modelling of SLJs under tension has been extensively examined and presented in the literature [19,20,21]. Based on the pioneering works of Volkersen [22] and Goland and Reissner [23] as well as the Hart Smith modified model [24], the effect of different factors such as adherends properties [25], stress fields developed [26], plasticity effects [27] and adhesive fillet [28], affecting the SLJs behavior, have also been studied and presented. 

Except for the above-mentioned parameters, a parameter of cardinal importance affecting the overall mechanical performance of adhesively bonded SLJs is the adherends’ surface pre-treatments by applying special techniques such as sol–gel, wet chemical, electrochemical deposition, and mussel-inspired chemistry [29]. Adherends’ surface pre-treatment is used, amongst others, for surface contaminants removal, surface roughness control, and surface wetting characteristics enhancement, thus leading to a better mechanical interlocking and/or adhesive/adherends’ chemical bonding. Generally, research on the electrochemical anodization of metals is conducted on standard plates provided by well-known suppliers. Anyhow, most of the metals that are used in the industrial sectors, such as aeronautics, shipbuilding, and automotive, are low-cost and alloyed; this is because the low-cost metals allow the acquisition of large parts. In the laboratory research, results are influenced by very small changes in the metal’s structure and composition (i.e., alloying or impurities). Thus, small differences in the metal’s structure (purity, density, and porosity) modify the anodization results. This makes it difficult to realize the transfer of the process from the lab to the industries/market. Investigations are required to manage the shifting of the new nano-functionalized joints to applications, which is the purpose of previous and the present studies [30]. 

The electrochemical anodization has several advantages, such as, simplicity in application and low-cost processing. Anodizing is an electrochemical process to passivate metals. The process is carried out by electrolysis (direct current channeling), in an acid solution bath, under strict conditions of control of the concentrations of the chemical components, the temperature, the density of the metal, etc. This process is difficult to compare with any other surface treatment, since during anodizing aluminum has an active role. As part of this process, a thin layer of aluminum oxidation is created that is fully integrated into the outer surface of the profiles, thus offering remarkable features. This method allows the formation of highly organized nanostructures (i.e., oxide nanotubes) and the functionalization of metallic surfaces for specific applications [31,32,33]. However, two main challenges are accounted for in this method. The first mentioned above is that it shows some difficulties when applied to industrial grade surfaces of complex geometry and big sizes, and the second one, is that depending on the metallic surfaces to be adhesively bonded together, a specific anodization recipe is needed. In our previous publications, the anodization method was used to both titanium-titanium [34] and aluminum-aluminum adhesively bonded SLJs with very promising results [35]. 

Although tensile-shear testing of SLJs is well documented and a lot of works on this topic can be found in literature, there are only few papers dealing with SLJ performance when under three-point bending conditions. However, since under real conditions, joints are loaded both in tension and bending, SLJs three-point bending testing is very important. Three-point bending, and tension loading are very similar in the way in which they affect the adhesive while the four-point bending does not cause failure because the metallic substrates yield before the joint fails [2]. In addition, due to the complex stress field developed within the joint, analytical models that have been developed to predict joints’ failure when under bending conditions, proved unsuccessful [36,37]. 

In the present work, the mechanical performance of single-lap aluminum adhesive joints using both non-anodized and anodized adherends were investigated and compared. Also, two different adhesives, the Araldite LY1564 resin and the eco-friendly Super Sap^®^ INR epoxy resin were used as adhesives. The later uses technology that reduces the environmental impact of the epoxies production, ultimately reducing the carbon footprint of the epoxy itself. Results of both tensile-shear testing and three-point bending showed that there is a considerable improvement of the joints’ mechanical performance when using nanostructured adherends, while in all cases, specimens with the eco-friendly adhesive showed superior shear performance as compared to respective specimens with Araldite adhesive. These results are very promising in replacing the standard epoxy resin adhesives with more eco-friendly adhesives, while at the same time, improving the aluminum SLJs mechanical behavior. 

## 2. Materials and Methods

### 2.1. Materials

Low-cost aluminum A1050 of 99.6% purity was purchased from Manousaridis Bros OE (Athens, Greece). Compared to most metals, it generally shows a ductile behavior, low mechanical strength, good electrical and thermal conductivity, low density (2.79 g/cm^3^), annealing temperature of 350–500 °C, and melting temperature of 650 °C (Table 1).

Two different types of resins were used. The first adhesive used was the Super Sap^®^ INR Epoxy combined with hardener Super Sap^®^ INF. As opposed to traditional epoxies that are composed primarily of petroleum-based materials, Super Sap^®^ formulations contain biobased renewable materials sourced as co-products or from waste streams of other industrial processes, such as wood pulp and bio-fuels production. These natural components have excellent elongation and exceptionally high adhesion properties. The Super Sap INR/INS epoxy system was prepared by mixing the two parts with a ratio of 100:33. The system underwent an initial cure at room temperature for 24 h and post cure at 120 °C for 2 h. The adhesive material used in the second epoxy resin system for the SLJs was a traditional resin type, Araldite LY 1564 (bisphenol A) combined with Aradur 2954 (cycloaliphatic polyamine) as curing agent at a ratio 100:35 parts by weight. The curing time was of 1 h at 80 °C followed by 8 h at 140 °C. The properties of the two resins used can be seen in Table 2.

### 2.2. Electrochemical Anodization of the Adherends

An alumina nanotube layer has been created on the surface of aluminum plates using the electrochemical anodization technique. The objective was to create a stronger interlock in the interphase between adherends and adhesive, as well as to improve the adherends’ surface roughness and increase their contact area with the adhesive. A graphite bar served as the cathode and the metallic plate served as the anode. The anodization was performed only in the joining region of the aluminum adherend. The electrodes were spaced approximately 2.5 to 3 cm apart. There are main parameters influencing the results of the aluminum anodization: (i) the electrolyte type, (ii) the anodization duration, and (iii) the applied voltage.

Using electrochemical anodization on the metal substrates it is possible, by applying a specific anodization recipe, to control the geometrical characteristics of nanotubes developed on the adherends surface and thus producing nanotubes of specific height and diameter, as well as surface density. Although this method is based on a trial-and-error procedure, once the recipe is found, then a pronounced increase in adhesive joints strength can be achieved. To find the best method for producing structured alumina nanotubes, many combinations of these variables were investigated until we reached the final anodizing formula, summarized in three steps. Prior to the anodization procedure, specimens were polished with sandpaper, starting from 320 grits for few seconds, followed by polishing with an intermediate sandpaper of 600 grits for 2 min, and ending with 5 min methanol bath cleaning. Some publications mention a two-steps anodization process applied on industrial alumina [41,42]. The protocol within the present investigation is formed of one preparation step and two steps of effective anodization. The three steps were found to be necessary to obtain the desired results. The protocol was previously developed as described in [35]: (i) Electropolishing: which is an exothermic process and it needs temperature control by freezing part of the electrolyte; (ii) Pre-anodization: allows a field-assisted dissolution of the oxide under a higher electric potential which is considered a prerequisite for the controlled formation of a porous alumina oxide; and (iii) Anodization: performed for an extended time (4 h) compared to the pre-anodization, which enables a guided self-building of the nanotube architecture. 

The electrolytes were sulfuric and oxalic acids-based. Sulfuric acid is used for Type III Hard Anodizing, also known as hard coat anodize, meant for components that are subject to extreme wear or highly corrosive environments to create a more durable coating needed in applications like blast shields, hinge mechanisms, valves, and joints [43]. Oxalic acid is commonly used in aluminum anodization and is appropriate for industrial aluminum too [41,42].

In the first anodization step, the electrolyte was a water solution of a half-frozen 3% (*w*/*w*) hydrofluoric acid (HF), while the anodization had a duration of 20 min and the applied voltage was 20 V. In step 2, the specimen was anodized in a solution of 15.12 mL H_2_C_2_O_4_ (oxalic acid) in 344.88 mL H_2_O and 40 mL (CH_2_OH)_2_ (ethylene glycol) for 10 min at a 40 V potential difference. Finally, in step 3, the aluminum was anodized in a solution of 15.12 mL H_2_C_2_O_4_ (oxalic acid) in 344.88 mL H_2_O and 40 mL (CH_2_OH)_2_ (ethylene glycol) for 240 min at a 60 V potential difference. Between the anodization steps, the samples were cleaned with deionized water, ethanol and dried before proceeding to the next step. After the anodization process, the adherends were put in ultra-sonic bath for 25 min, in a solution of H_3_PO_4_ 3 wt.%, to remove the chemical traces of the electrolytes [35]. The ultrasonication was applied as described after the last anodization step and was a pore widening step. 

### 2.3. Specimens’ Manufacturing

**Figure 2:**
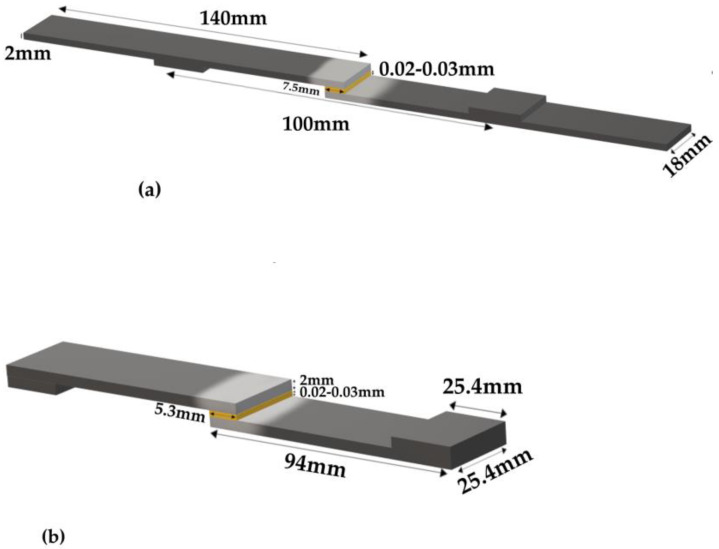
Adhesive single-lap joint specimens (**a**) for three-point bending test (**b**) for tensile test.

**Figure 3:**
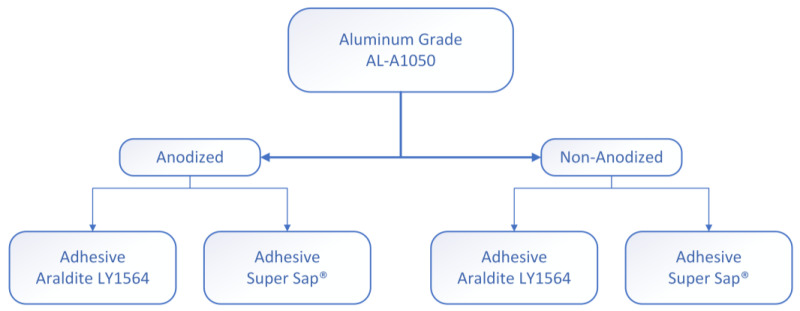
The four different types of single-lap joints manufactured and tested.

Two types of specimens were manufactured for each test that was conducted, due to the different dimensional requirements. The joints designed for tensile testing were prepared according to the ASTM-D1002. The aluminum adherends had dimensions of 94 × 25.4 × 2 mm as shown in Figure 2a. The aluminum adherends for the three-point bending test had different dimensions of 140 × 18 × 2 mm as shown in Figure 2b. For each test, four different combinations of anodized/non-anodized aluminum adherends bonded with Araldite/Super Sap adhesives were manufactured (Figure 3).

The preparation of the Araldite LY-1564/Aradure 2954 epoxy system began by mixing them at a ratio of 100:35, then stirring the mixture for a few minutes and putting it in an air-vacuum chamber for 5–7 min. After that, the epoxy adhesive was applied to the overlap area, with the overlap lengths presented in Figure 2 and the adherents were bonded under controlled pressure at room temperature conditions. Subsequently, the SLJs were put in the oven for 1 h at 80 °C and then for 8 h at 140 °C. The Super Sap INR/INS epoxy system was prepared by mixing the two parts at a ratio of 100:33, same precured process had been followed except that the specimens were left for 24 h in ambient conditions and then were put in the oven for 2 h at 120 °C. After the adhesive was cured the specimens were kept in air-sealed plastic bags.

### 2.4. Surface Analysis and Mechanical Characterization

A Scanning Electron Microscope (SEM) device, Model Zeiss SUPRA 35VP (Jena, Germany) was used to observe the adherends’ contact area micro/nanostructure.

The apparent shear strength of the adhesive was determined through tensile loading of the joint, as indicated by ASTM D1002-01, using an Instron 8872-25 kN servohydraulic (High Wycombe, UK) universal mechanical testing machine. In all cases, a constant crosshead speed of 1 mm/min was applied. Based on the maximum tensile forces recorded for each tested joint, the adhesive shear strength *τ* was calculated as the maximum shear stress attained in an adhesive layer using the formula:

(1) $$\tau =\frac{P}{w\cdot l_{0}}$$

where *τ* is the adhesive shear strength, *P* is the load, *w* is the width, and *l*_0_ is the overlap length. Five or more specimens per type (i.e., anodized/non-anodized and Araldite/Super Sap) were tested to ensure the repeatability of the results.

**Figure 4:**
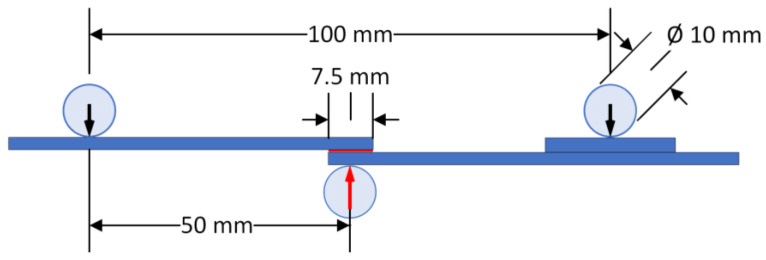
Schematic representation of the joints flexural loading as applied.

**Figure 5:**
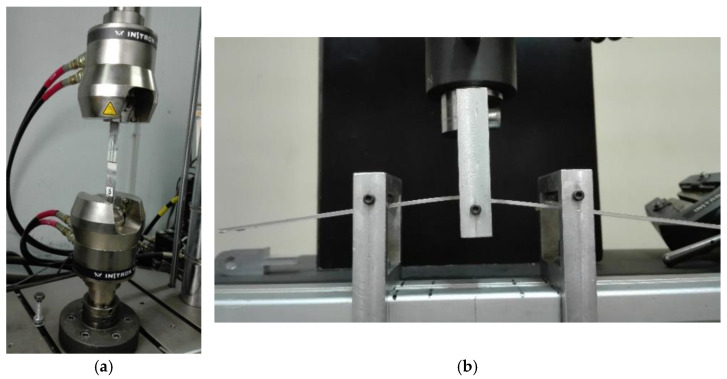
Experimental setup of a single-lap joint for (**a**) tensile-shear loading, (**b**) flexural loading.

The flexural behavior of the manufactured SLJs was studied through a series of quasi-static three-point bending tests using an Instron 4301 (High Wycombe, UK) universal mechanical testing machine. A schematic representation of the three-point bending test as executed in all types of joints tested is shown in Figure 4. The tests were performed at room temperature with a constant crosshead speed of 1 mm/min. Five or more specimens per type (i.e., anodized/non-anodized and Araldite/Super Sap) were tested to ensure the repeatability of the results. The experimental setups used are shown in Figure 5.

### 2.5. Data Analysis

The experimental results for the tensile-shear tests were obtained as described in the previous sub-chapter and the maximum apparent shear strength was calculated using Equation (1). The individual specimens’ values for each case (i.e., anodized/non-anodized and Araldite/Super Sap) were used to calculate the mean maximum shear strength, the standard deviation, and the coefficient of variation of these experimental values. The coefficient of variation never exceeded 10%.

For the three-point bending tests the same procedure was followed. The mean values, the standard deviation, and the coefficient of variation for failure load, failure deflection, and load-deflection slope were calculated.

## 3. Results and Discussion

### 3.1. Scanning Electron Microscopy

In the present work, a specific protocol of hard anodization of aluminum plates was applied for the development of surface alumina nanotubes layers. The aluminum plates are intended to be used subsequently for the manufacture of adhesive SLJs. The main anodizing parameters affecting the nanotubes size and morphology are the aluminum alloy composition, the anodizing voltage, the type of electrolyte, and the duration of the anodization process. Therefore, the anodization process is a highly controlled oxidation phenomenon. For the present investigation, electrolytes and voltage intensities were combined until the ideal recipe for the employed aluminum was found. It was previously stated that anodizing in sulfuric acid results in small pore sizes and interpore distances compared to the ones obtained by anodizing in phosphoric and oxalic acids [44]. Three-step anodization method has been utilized before to prepare anodic alumina templates with various pore morphologies (e.g., arched-shape, tree-like, branched-shape) and tunable interpore distances and it was affirmed that such structures are not found within the more traditional alumina templates fabricated by a two-step anodization of aluminum films [45]. Recently, it was found that the optimal etching effect on alumina is obtained with 1 wt% HF, which is the reason why a three-step anodization procedure has been chosen [46]. However, the previously mentioned concentration of hydrofluoric acid was low for the type of aluminum used in the present investigation and consequently it needed to be adjusted to 3 wt%.

**Figure 6:**
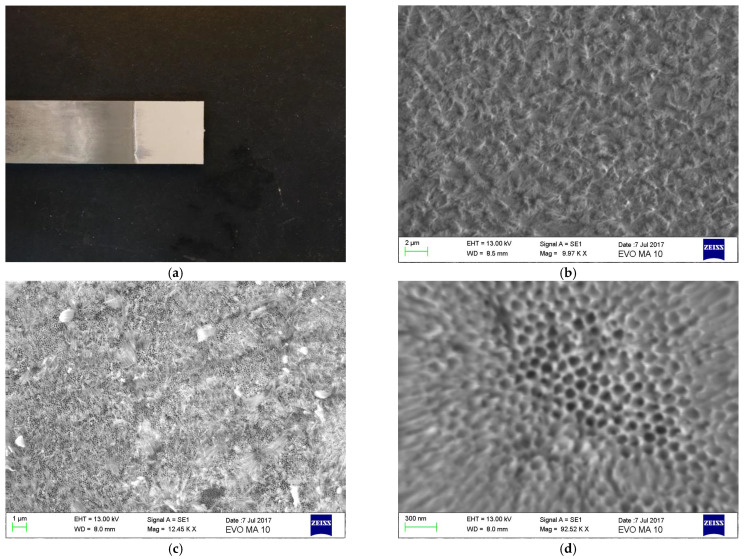
Micrographs of aluminum plate: (**a**) adherend after anodic treatment, (**b**) SEM after the second anodization step, (**c**) SEM after the third anodization step, without ultrasonication and (**d**) SEM after ultrasonication.

The aluminum plates topography at the different protocol anodization stages is shown in Figure 6. To achieve both, chemical traces elimination and pore widening, ultrasonication of anodized aluminum plates was applied in a 3 wt% H_3_PO_4_ water solution. Prior to applying ultrasonication, nanotubes were not clearly observed (Figure 6c).

Figure 6b shows the obtained roughness after the second anodization step (20 V for 20 min) is completed. After the third anodization step is accomplished, a continuous distribution of pores may be observed on the entire analyzed surface, with pore diameter around 80–90 nm (Figure 6c). However, upon the completion of the last stage of anodization pores may not be clearly observed (Figure 6c). After ultrasonication the impurities layer is removed, and pores are widened (Figure 6d).

These findings prove that parameters such as electrolyte type, applied voltage and electrochemical anodization duration strongly affect the nanotubes size and morphology while, at the same time, they are in complete agreement with respective results found in Araoyinbo et al. [47]. Our findings are in agreement with other bibliographic studies; it has been stated that the formation of the aluminum oxide layer and the control of the anodization process is a challenge, since the presence of alloying elements affects not only the rate of oxide growth but also the microstructure of the anodic film. Furthermore, it was found that pore circularity and regularity of pore arrangement in AAO membranes formed on the AA1050 alloy were always worse than those observed on the pure Al substrate. The structural features, such as pore diameter, interpore distance, wall thickness, barrier layer thickness, porosity, and pore density of porous anodic alumina formed on AA1050 are a little different from those obtained for high purity aluminum [41]. That is why an adapted protocol consisting of one preparation procedure by electropolishing and two anodization steps are needed.

### 3.2. Tension-Shear Testing

In the present paragraph, results for the tension-shear tests of all types of joins manufactured, are presented.

**Figure 7:**
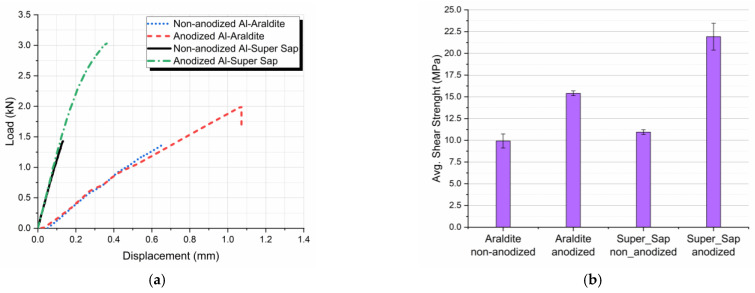
Effect of aluminum adherends anodization on the aluminum single-lap joints bonded with Araldite and Super Sap adhesive tensile-shear behavior; (**a**) load-displacement curves, (**b**) bar diagram for the joints’ average shear strength values.

Figure 7a shows load-displacement curves for joints with non-anodized and anodized aluminum adherends using Araldite and Super Sap adhesive. Average shear strengths values were found to be 9.92 MPa and 15.4 MPa respectively for the Araldite and 10.94 MPa and 21.92 MPa respectively for the Super Sap adhesive, as presented in Figure 7b.

To better quantify the results, average shear strength values for all four types of joints manufactured and tested in tension-shear, are given in Table 3. These values show separately both the effect of anodization and the adhesive type on the shear lap joints’ shear strength values. Thus, by anodizing the aluminum in the Araldite joints, an increase of 55.5% in shear strength was achieved, while for the Super Sap joints, an increase of 100.9% in shear strength was obtained for the anodized aluminum joints. 

However, in Table 3 the results are also cross-examined to quantify the adhesives effect on the joint. According to the results shown, Super Sap joints’ shear strength are higher than Araldite joints’ respective values, especially in the case of the anodized specimens, where an increase on the order of 42.2% was observed. 

From the results presented in Table 3 it follows that:

1. For both types of adhesives applied, aluminum anodization resulted in shear strength enhancement.
2. The use of the eco-friendly adhesive resulted in a superior shear behavior of the joints as compared with the joints where Araldite adhesive was used.
3. The maximum tensile-shear strength was achieved when using both anodized aluminum adherends and the eco-friendly epoxy adhesive.

The above-mentioned findings can be explained through the parameters affecting the shear strength. Despite its simplicity, tension shear testing is characterized by several drawbacks. To mention, upon loading, a differential shear effect is observed, according to which a non-uniform stress and strain distribution in the adhesive-adherend interfacial area is developed, potentially affecting the results, and leading to incorrect values concerning the adhesive strength. More precisely, as the joint is under tensile loading, adherend tensile stress attains a maximum value at the overlap edge which is closer to the side of the applied load, decreasing subsequently, tending to zero at the other overlap edge. The same variation is observed for the adhesive-adherend interfacial strain leading to the development of complicated non-uniform interfacial stress and strain fields. These non-uniform stress and strain fields over the bond area result in the development of shear stress concentrations at the overlap edges, disturbing the reported shear stress experimental value, which is lower than the true ultimate strength of the adhesive. In addition, even if all measures have been taken for the development of a strong adhesive-adherend adhesion, environmental conditions, such as atmospheric moisture, strongly degrade the adhesion bond and shorten the adhesive joint life [48].

Adhesive-adherend adhesion is strongly affected by adherends’ surface roughness since roughness changes the surface energy of the materials in contact. The term roughness refers to the deviation of a surface from its mean plane, and it is characterized by statistical parameters such as the variance of height, slope, and curvature. However, these parameters strongly depend on the roughness measuring instrument resolution, as well as on the measuring direction. As a result, according to its definition, surface roughness is a random non-stationary and multiscale process. Adherends surface topography greatly affects the load-carrying capability of adhesive joints. 

In their study of the relationship between adherend surface roughness and adhesive bond strength, Ghumatkar et al. [49] discovered that there is an ideal surface roughness for a maximum bonding strength and that the roughness range depends on the adherend material, while joint strength changes are associated Also, they came to the conclusion that there are other factors contributing to the increase in strength, hence a simple connection with surface roughness is insufficient to predict joint performance.

Cho et al. [50] studied the impact of surface roughness on adhesive strength of the heat resistant adhesive RTV88 by experiments and parameter analysis. To more clearly describe how surface roughness affects adhesive strength, the terms effective area, peel failure area, and cohesive failure area were established. In general, when surface roughness increases, the effective area, cohesive failure area, and shear strength all increase. Although the effective area grows as surface roughness increases, the shear strength decreases because, as surface roughness reaches a critical value, the cohesive failure area reduces.

Moreover, Pereira et al. [51] have reported for their aluminum alloy adhesive lap joints that the decrease in surface roughness was found to increase the shear strength of SLJs.

These seemingly contradictory results found in the literature lead to the conclusion that, with increasing surface roughness, an initial increase in shear strength is obtained up to a specific surface roughness value where shear strength attains its maximum value, while for values beyond this critical roughness value, a decrease in shear strength is obtained. The critical surface roughness value depends on the adherends-adhesive materials combination as well as on the surface pretreatment type.

Based on the above-mentioned findings, our results show that the degree of adherends surface roughness achieved by electrochemical anodization is close to the so-called critical roughness value, leading to a high value of the anodized SLJs shear strength as compared to the non-anodized joints. In addition, since the overall shear behavior of the joints depends not only on the adhesive type applied alone but on the adherend-adhesive material combination and the surface roughness as well, the application of the eco-friendly adhesive better fulfills the requirements for a high-value shear strength according to the results found. As a result, shear strength values for the non-anodized joints are close for both adhesives, while in the case of anodized joints, Super Sup adhesive shows higher shear strength values as compared with respective Araldite adhesive joints.

### 3.3. Three-Point Bending

The flexural behavior of all types of aluminum SLJs is evaluated by three-point bending tests under quasi-static conditions. 

**Figure 8:**
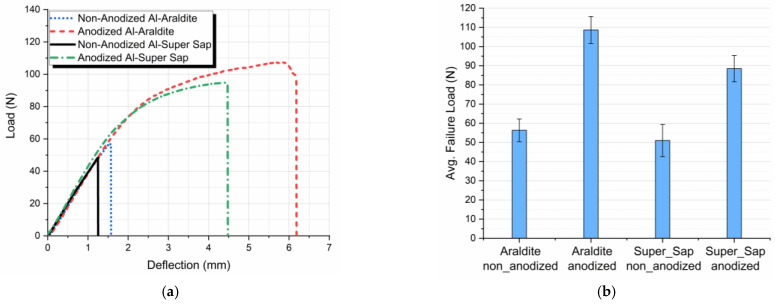
Effect of aluminum adherends anodization on the aluminum single-lap joints bonded with Araldite and Super Sap adhesive three-point bending behavior; (**a**) load-deflection curve, (**b**) bar diagram for the joints’ average flexural failure load.

All results were plotted in terms of applied load versus center deflection of the specimens (Figure 8a). All specimens had the same geometry and were tested under the same crosshead speed, thus making it possible to superimpose the load/displacement plots for each group of samples. This allowed a more accurate comparison of the resulting curves. 

The effect of anodization on the bending performance of the different types of joints can be deduced from the typical load-deflection curves shown in Figure 8a for non-anodized and anodized aluminum SLJs bonded with both Araldite and Super Sap adhesive.

From these curves it can be observed that non-anodized Al-Araldite SLJs show a linear elastic behavior which is independent on the adhesive type applied. In contrast, anodized aluminum joints show a ductile behavior with a higher ultimate load values and higher failure deflection as compared to the non-anodized aluminum joints. Thus, anodization always results to a better flexural behavior of the joints. 

The observed behavior can be explained in combination with the adhesion mechanism described already in the previous paragraph where the anodized joints exhibited a stronger bond between adhesive and adherend. Thus, in the case of anodized joints, as the joint is under flexural load, the adhesion bond, due to its strength, resists to bending while the aluminum adherends are deformed leading to deformation values within their plasticity range, increasing thus the obtained overall joint failure load. The adherends plastic deformation observed during the joint flexural loading can be observed in Figure 5b. 

Also, the effect of adhesive type used on the bending performance of the different types of joints can be deduced from the typical load-deflection curves shown in Figure 8a. From these curves it follows that joints bonded with Araldite adhesive show a superior bending performance for both anodized and non-anodized aluminum adherends. Finally, the slope in the linear region of the force-deflection response was measured and its value was found independent to both adhesive type and adherend surface treatment. This is because the slope in the linear region of the force-deflection response mainly depends on the adherend stiffness. 

At this point, it is worth mentioning that there is a significant effect of anodization on the flexural failure load of the joints bonded with both adhesives. More precisely, in the case of joints bonded with Araldite adhesive, anodization of aluminum adherends resulted in an increase of 92.9% in the flexural failure load while the respective increase for the joints bonded with Super Sap adhesive was 73.5%. Comparing the effect of the adhesive type applied under the same aluminum surface treatment conditions, joints bonded with Araldite adhesive show a small increase in failure load values as compared to the joints bonded with Super Sap adhesive. Detailed numerical results and comparisons between the different types of joints with respect to the average failure load are given in Table 4.

**Figure 9:**
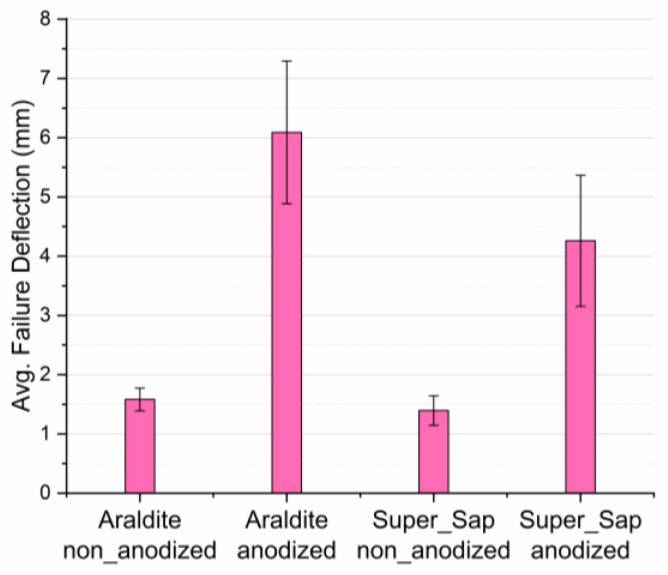
Bar diagram for the average flexural failure deflection values of all four types of joints manufactured and tested.

Next, by comparing the failure deflections of the different types of joints, it can be concluded that adherends’ anodization greatly affects their values. More precisely, in the case of joints bonded with Araldite, anodized joints show an increase of 283% in failure deflection values while a respective increase of 206% has been observed in joints bonded with Super Sap adhesive. On the other hand, the type of adhesive used plays an inferior role on the failure deflection values of the joints tested. Experimental results for failure deflection showed high standard deviations between the values of the numerous specimens tested and this is attributed to the fact that failure deflection is sensitive to a great number of manufacturing and experimental parameters such as void existence within the adherend-adhesive interface/interphase area, surface uniformity of nanotubes developed in the contact area, pressure applied uniformity during joint manufacturing, etc. Detailed results for the average failure deflection values are shown in both Figure 9 and Table 5.

**Figure 10:**
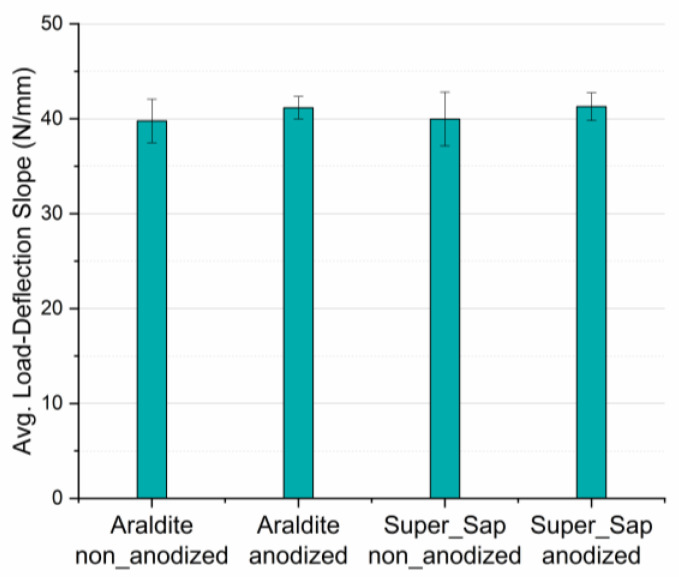
Bar diagram for the average load-deflection curves initial slope values of all the four types of joints manufactured and tested.

Finally, since the slope in the linear region of the force-deflection response is a measure of the joint stiffness when under bending conditions, slope values for all types of joints were measured and analyzed. It was found that slope values are independent on adherends anodization and type of adhesive applied, having an almost constant value of 40 N/mm in all types of joints. This behavior shows that joint stiffness mainly depends solely on the adherends material type, and it is not affected by the adhesive applied, nor by the adherends surface treatment. Detailed numerical results are shown in both Figure 10 and Table 6.

## 4. Conclusions

In the present work, in an effort of replacing Araldite adhesive with an eco-friendly one, aluminum SLJs were manufactured and tested under tensile-shear testing and three-point bending. The effect of two parameters combinations such as the adhesive type applied and the aluminum adherends surface treatment by means of electrochemical anodization were studied. The main conclusions are as follows:

- For both types of adhesives applied, aluminum anodization resulted in shear strength enhancement,
- The use of the eco-friendly adhesive resulted in a superior shear behavior of the joints as compared with the joints where Araldite adhesive was used.
- A maximum tensile-shear strength enhancement of 42.2% was achieved when using anodized aluminum adherends bonded with the eco-friendly epoxy.
- In joints bonded with Araldite adhesive, anodization of aluminum adherends resulted to a considerable increase of 92.9% in the flexural failure load as compared with the non-anodized ones, while the respective increase for the joints bonded with Super Sap adhesive was 73.5%.
- The findings clearly showed that using anodized aluminum adherends bonded with the eco-friendly Super Sap adhesive we can achieve better mechanical performance of the aluminum single-lap joints as compared with the harmful for the environment aluminum single-lap joints bonded with Araldite epoxy adhesive, thus decreasing the environmental footprint.

## Figures and Tables

**Table 1:** Aluminum A1050 properties [38].

Property	Value
Density (Kg/m^3^)	2.79
Modulus of Elasticity (GPa)	73
Tensile Strength (MPa)	75
Proof Stress 0.2% (MPa)	35
Fatigue Strength 50 mil. Cycles (MPa)	20
Shear Strength (MPa)	50
Hardness Vickers (HV)	22
Hardness Brinell	20
Elongation 50 mm (%)	32
Melting Point (°C)	640
Thermal Conductivity (W/m∙K)	121–193
Thermal Expansion	23.1 × 10^−6^ grad^−1^

**Table 2:** Applied adhesives properties [39,40].

Property	Super Sap INR/INS	Araldite LY1564/Aradur 2954
Viscosity at 25 °C (mPas)	2200/25	1200–1400/70–120
Density (g/cm^3^)	1.1 (mixed)	1.1–1.2/0.94–0.95
Mix ratio (PBW)	100:33	100:35
Cure cycle	24 h at 25 °C + 2 h at 120 °C	1 h at 80 °C + 8 h at 140 °C
Tensile Modulus (GPa)	3.38 ^1^	2.55–2.65 ^4^
Tensile Strength (MPa)	68.9 ^1^	71–77 ^4^
Elongation (%)	3–4 ^1^	4.5–5.5 ^4^
Flexural Modulus (GPa)	2.62 ^2^	2.6–2.8 ^5^
Flexural Strength (MPa)	105.5 ^2^	120–124 ^5^
Ultimate Tg by DSC (°C)	104.4 ^3^	148 ^6^

^1^ ASTM D638; ^2^ ASTM D790; ^3^ ASTM D3418; ^4^ ISO 527; ^5^ ISO 178; ^6^ IEC 1006, 10 K/min.

**Table 3:** Comparison of the average shear strength values for the different types of adhesive aluminum single-lap joints manufactured and tested.

Type of Joint (Anodization Effect)	Shear Strength (MPa)	Difference (%)	Type of Joint (Adhesive Effect)	Shear Strength (MPa)	Difference (%)
Araldite non-anodized	9.9	55.5	Araldite non-anodized	9.9	10.1
Araldite anodized	15.4		Super Sap non-anodized	10.9	
Super Sap non-anodized	10.9	100.9	Araldite anodized	15.4	42.2
Super Sap anodized	21.9		Super Sap anodized	21.9	

**Table 4:** Comparison of average failure load values for the different types of Aluminum single-lap joints.

Type of Joint (Anodization Effect)	Failure Load (N)	Difference (%)	Type of Joint (Adhesive Effect)	Failure Load (N)	Difference (%)
Araldite non-anodized	56.3	92.9	Araldite non-anodized	56.32	−9.4
Araldite anodized	108.6		Super Sap non-anodized	51.00	
Super Sap non-anodized	51.0	73.5	Araldite anodized	108.6	−18.5
Super Sap anodized	88.5		Super Sap anodized	88.5	

**Table 5:** Comparison of three-point bending average failure deflection values for the different types of aluminum single-lap joints.

Type of Joint (Anodization Effect)	Failure Deflection (mm)	Difference (%)	Type of Joint (Adhesive Effect)	Failure Deflection (mm)	Difference (%)
Araldite non-anodized	1.59	283.0	Araldite non-anodized	1.59	−12.6
Araldite anodized	6.09		Super Sap non-anodized	1.39	
Super Sap non-anodized	1.39	206.5	Araldite anodized	6.09	−30.0
Super Sap anodized	4.26		Super Sap anodized	4.26	

**Table 6:** Comparison of the three-point bending average load-deflection curves initial slope values for the different types of aluminum single-lap joints.

Type of Joint (Anodization Effect)	Load-Deflection Slope (N/mm)	Difference (%)	Type of Joint (Adhesive Effect)	Load-Deflection Slope (N/mm)	Difference (%)
Araldite non-anodized	39.8	3.5	Araldite non-anodized	39.8	0.5
Araldite anodized	41.2		Super Sap non-anodized	40	
Super Sap non-anodized	40	3.3	Araldite anodized	41.2	0.2
Super Sap anodized	41.3		Super Sap anodized	41.3	

## Data Availability

The data presented in this study are available in manuscript.
